# Methicillin-Resistant *Staphylococcus aureus* USA400 Clone, Italy

**DOI:** 10.3201/eid1506.081632

**Published:** 2009-06

**Authors:** Carla Vignaroli, Pietro E. Varaldo, Alessandro Camporese

**Affiliations:** Polytechnic University of Marche, Ancona, Italy (C. Vignaroli, P.E. Varaldo); Santa Maria degli Angeli Regional Hospital, Pordenone, Italy (A. Camporese)

**Keywords:** Staphylococcus aureus, staphylococci, bacteria, antimicrobial resistance, community-associated infections, bacterial typing, Panton-Valentine leukocidin, Italy, letter

**To the Editor:** In the past 30 years, methicillin-resistant *Staphylococcus aureus* (MRSA) has been the leading cause of nosocomial infections throughout the world. Healthcare-associated MRSA (HA-MRSA) isolates are resistant to multiple antimicrobial drugs. This resistance severely hampers treatment options. During the past decade, MRSA isolates have also emerged as major pathogens in the community, first in the United States and later worldwide. Community-associated MRSA (CA-MRSA) isolates are usually more susceptible to antimicrobial drugs but are more virulent than HA-MRSA isolates. Among various determinants involved in the pathogenesis of CA-MRSA infections, special attention has been focused on the Panton-Valentine leukocidin (PVL), which has a strong epidemiologic link with CA-MRSA clones (*1*).

It has been suggested that CA-MRSA might move to healthcare settings, blurring the line between HA- and CA-MRSA (*2*). Nevertheless, CA-MRSA isolates are increasingly being reported as pathogens in the general population in persons with no risk factors for HA-MRSA acquisition. These pathogens are generally associated with skin and soft tissue infections, but also with more severe infections such as necrotizing pneumonia or septicemia. CA-MRSA strains usually harbor a staphylococcal cassette chromosome (SCC) *mec* (type IV or V) that is smaller than the type I–III SCC*mec* elements commonly found in HA-MRSA strains. To date, 5 major CA-MRSA clonal lineages from diverse genetic backgrounds have been recognized by pulsed-field gel electrophoresis and multilocus sequence typing; certain clones predominate in specific areas of the world (*1*).

The most common lineages in the United States are sequence type (ST) 1 (USA400) and ST8 (USA300), which usually carry type IV SCC*mec* and PVL-encoding genes. Over the past few years, ST8 (USA300) has become predominant in the United States (*3*), also emerging as a major cause of nosocomial infections (*4*). In Europe, data are more limited, but the situation appears to be more varied: the predominant CA-MRSA clonal lineage is ST80 (*5*), although single cases or small clusters caused by ST8 (USA300) have increasingly been reported (*6**–**8*). In contrast, the ST1 (USA400) clone is still rare in Europe (*9*,*10*). We describe the importation of ST1 (USA400) into Italy and its isolation in the country. The organism was isolated from an Italian woman with a skin infection that she contracted in the United States.

In late November 2007, a 36-year-old Italian woman was seen at Pordenone Hospital (northeastern Italy) for spider-bite–like skin lesions on the face, characterized by rapid evolution to furuncles and small abscesses. The infection had started ≈1 month earlier in California, where she had spent several months on business (wine import-export), and where she had been treated empirically with amoxicillin/clavulanate for 10 days (1 g, 3×/day), with no clinical improvement.

Culture of the pus from the abscesses yielded an MRSA isolate that was resistant to oxacillin and susceptible to all non-β-lactam antimicrobial drugs tested by Vitek 2 AST-P536 card (bioMérieux, Marcy l’Etoile, France). Such a particular susceptibility pattern and the community origin of the infection prompted molecular investigation and typing by established methods, which confirmed the isolate to be CA-MRSA and identified it as belonging to the USA400 clone (ST1, type IVa SCC*mec*, presence of PVL genes, *agr* type III, *spa* type t128). Notably, t128 is the *spa* type found in MW2, the highly virulent prototype strain of USA400. Treatment with oral levofloxacin for 7 days (500 mg, 1×/day) led to complete resolution of the infection. After more than a year, the patient has experienced no recurrences.

All 3 previously reported cases of CA-MRSA infection in Italy were caused by type IV SCC*mec*, PVL-positive strains, none of which, however, belonged to the ST80 clonal lineage that predominates in Europe (*7*). The first case (in 2005) was a necrotizing pneumonia caused by an ST30 isolate; the 2 other cases (2006) were severe invasive sepsis and a neck abscess, both caused by ST8 (USA300) isolates. The case we note here documents the importation of a US pathogen into a country in Europe, from an area where the pathogen is widespread and has been highly virulent since the late 1990s, to an area where its penetration in the past has been poor.

## References

[1] Diep BA, Otto M. The role of virulence determinants in community-associated MRSA pathogenesis. Trends Microbiol. 2008;16:361–9. 10.1016/j.tim.2008.05.002 18585915 PMC2778837

[2] Tenover F. Community-associated methicillin-resistant *Staphylococcus aureus*: it’s not just in communities anymore. Clin Microbiol Newsl. 2006;28:33–6. 10.1016/j.clinmicnews.2006.02.001

[3] King MD, Humphrey BJ, Wang YF, Kourbatova EV, Ray SM, Blumberg HM. Emergence of community-acquired methicillin-resistant *Staphylococcus aureus* USA 300 clone as the predominant cause of skin and soft tissue infections. Ann Intern Med. 2006;144:309–17. 16520471 10.7326/0003-4819-144-5-200603070-00005

[4] Seybold U, Kourbatova EV, Johnson JG, Halvosa SJ, Wang YF, King MD, Emergence of community-associated methicillin-resistant *Staphylococcus aureus* USA300 genotype as a major cause of health care-associated blood stream infections. Clin Infect Dis. 2006;42:647–56. 10.1086/499815 16447110

[5] Deurenberg RH, Vink C, Kalenic S, Friedrich AW, Bruggeman CA, Stobberingh EE. The molecular evolution of methicillin-resistant *Staphylococcus aureus.* Clin Microbiol Infect. 2007;13:222–35. 10.1111/j.1469-0691.2006.01573.x 17391376

[6] Witte W, Strommenger B, Cuny C, Heuck D, Nuebel U. Methicillin-resistant *Staphylococcus aureus* containing the Panton-Valentine leucocidin gene in Germany in 2005 and 2006. J Antimicrob Chemother. 2007;60:1258–63. 10.1093/jac/dkm384 17934203

[7] Tinelli M, Pantosti A, Lusardi C, Vimercati M, Monaco M. First detected case of community-acquired methicillin-resistant *Staphylococcus aureus* skin and soft tissue infection in Italy. Euro Surveill. 2007;12:E070412.1. 10.2807/esw.12.15.03173-en 17439803

[8] Ruppitsch W, Stoger A, Schmid D, Fretz R, Indra A, Allerberger F, Occurrence of the USA300 community-acquired Staphylococcus aureus clone in Austria. Euro Surveill. 2007;12:E071025.1. 10.2807/esw.12.43.03294-en 17997911

[9] Witte W, Braulke C, Cuny C, Strommenger B, Werner G, Heuck D, Emergence of methicillin-resistant *Staphylococcus aureus* with Panton-Valentine leukocidin genes in central Europe. Eur J Clin Microbiol Infect Dis. 2005;24:1–5. 10.1007/s10096-004-1262-x 15599784

[10] Harbarth S, François P, Schrenzel J, Fankhauser-Rodriguez C, Hugonnet S, Koessler T, Community-associated methicillin-resistant *Staphylococcus aureus*, Switzerland. Emerg Infect Dis. 2005;11:962–5. 15963298 10.3201/eid1106.041308 PMC3367580

